# Modified microwave method for the synthesis of visible light-responsive TiO_2_/MWCNTs nanocatalysts

**DOI:** 10.1186/1556-276X-8-346

**Published:** 2013-08-06

**Authors:** Firas K Mohamad Alosfur, Mohammad Hafizuddin Haji Jumali, Shahidan Radiman, Noor J Ridha, Mohd Ambar Yarmo, Akrajas Ali Umar

**Affiliations:** 1 School of Applied Physics, Faculty Science and Technology, Universiti Kebangsaan Malaysia, Bangi, Selangor 43600, Malaysia; 2 School of Chemical Sciences and Food Technology, Faculty of Science and Technology, Universiti Kebangsaan Malaysia (UKM), Bangi, Selangor 43600, Malaysia; 3 Institute of Microengineering and Nanoelectronic (IMEN), Universiti Kebangsaan Malaysia, Bangi, Selangor 43600, Malaysia; 4 Physics Department, Science College, Baghdad University, Baghdad, Iraq

**Keywords:** Microwave, TiO_2_/MWCNTs, Hybrid nanocatalysts, Photocatalytic

## Abstract

Recently, TiO_2_/multi-walled carbon nanotube (MWCNT) hybrid nanocatalysts have been a subject of high interest due to their excellent structures, large surface areas and peculiar optical properties, which enhance their photocatalytic performance. In this work, a modified microwave technique was used to rapidly synthesise a TiO_2_/MWCNT nanocatalyst with a large surface area. X-ray powder diffraction, field-emission scanning electron microscopy, transmission electron microscopy and Brunauer-Emmett-Teller measurements were used to characterise the structure, morphology and the surface area of the sample. The photocatalytic activity of the hybrid nanocatalysts was evaluated through a comparison of the degradation of methylene blue dye under irradiation with ultraviolet and visible light. The results showed that the TiO_2_/MWCNT hybrid nanocatalysts degraded 34.9% of the methylene blue (MB) under irradiation with ultraviolet light, whereas 96.3% of the MB was degraded under irradiation with visible light.

## Background

Industrial advancements over the past several decades have led to an upsurge in the rate of water consumption. Due to the scarcity of clean water resources, the recycling of water via the elimination of extremely coloured wastewater has become important. Different methods, such as adsorption [1], oxidation [2], reduction [3] and anaerobic treatments [4], have been developed for the elimination of dyes from effluents. Unfortunately, these methods have several disadvantages [5-7], which have triggered interest among scientists in developing a method to decompose the undesirable organic compounds, such as dyes, via photocatalytic processes using the semiconductor degradation method [8-10]. This method offers several advantages, such as being simpler, cheaper and cleaner. Hence, this method is acknowledged as being a ‘greener’ technology for the elimination of toxic organic and inorganic pollutants from wastewater at ambient temperature and pressure [11-13].

Titania (TiO_2_) nanoparticles have been identified as a suitable material for the removal of dyes from effluents. However, due to its wide bandgap (3.2 eV), TiO_2_ exhibits photocatalytic activation only under UV irradiation (*λ* ≤ 384 nm), which accounts for only 7% of the total solar energy [14]. Several methods have been suggested to improve the photocatalytic activity of TiO_2_ in the visible light range [15-17]. Unfortunately, these methods involve compounds that are either thermally unstable, difficult to modify or even toxic [18].

Recently, there is growing interest in the hybridisation of TiO_2_ and carbon-based nanostructures, namely single-walled carbon nanotubes (SWCNTs) [19,20], multi-walled carbon nanotubes (MWCNT) [21,22] and graphene [23,24], as an attempt to improve the photocatalytic activity of TiO_2_. This improvement was attributed to three main factors namely the enlarged absorption region of TiO_2_[25-27], enhanced electronic transfer and thus reduced electron accumulation in TiO_2_ nanoparticles [28,29] and extremely high surface area [30,31].

The TiO_2_ nanoparticle attachment to MWCNTs can be prepared using different methods, such as hydrothermal [32], sol-gel [22] or electrochemical [33] methods. However, most of these methods require long preparation times (several hours or a day), involve multiple steps and have high thermal costs, which often result in structural damage in the MWCNTs. Thus, there is a need to develop an easier and faster method for their synthesis.

The synthesis of nanostructured materials via microwave irradiation has been reported to be an effective technique [34-36]. This technique offers several advantages, such as simple and fast synthesis procedures, improved reaction kinetics, uniform heat distribution and minimal structural damage [37]. In this work, a novel technology is presented for the synthesis of a hybrid photocatalytic material with greater photocatalytic activities and a wider spectral response range using a modified microwave method. Our previous report detailed the synthesis and optical properties of TiO_2_/MWCNTs hybrid nanocatalysts using a modified microwave method [38]. The results showed an enhancement in the optical absorbance, which was shifted from the UV to the visible light region after the MWCNTs were decorated with TiO_2_ nanoparticles. Here, we demonstrate our extended effort to extensively study the structural properties and, in particular, the photocatalytic application of these hybrid nanocatalysts.

## Methods

A modified microwave method was used to synthesise the TiO_2_/MWCNTs hybrid nanocatalysts. Initially, a 3.5-cm hole was drilled through the top of a household microwave oven. A reflux condenser was subsequently installed in the microwave oven to enable continuous synthesis at ambient pressures. Since the microwave has a wavelength of 12 cm, there will be no escaped radiation through the hole. As additional protection purpose, the microwave was operated inside a fume hood. Commercial MWCNTs (Cheap Tubes Inc., Brattleboro, VT, USA) with an outer diameter of 10 to 30 nm, an inner diameter of 5 nm, a surface area of 110 m^2^/g and lengths up to 50 μm were used in this work. Due to electrostatic interactions and van der Waals forces between the individual nanotubes, the MWCNTs exhibit a strong tendency to agglomerate. This agglomeration leads to poor solubility of the MWCNTs in most aqueous and organic solvents. Thus, to achieve a stable aqueous suspension of MWCNTs, functionalisation processes are necessary due to the presence of a large amount of functional groups on the nanotubes' surface. The presence of these functional groups on the MWCNTs' surface imparts negative charges and thus generates repulsion forces, which inhibit agglomeration. These negative charges can also function as anchor sites and thereby enable the *in situ* attachment of synthesised nanoparticles onto the MWCNTs' surface.

For this purpose, the MWCNTs were first functionalised by being sonicated for 3 h in a 65% solution of concentrated HNO_3_. The suspended MWCNTs were then placed in the modified microwave oven (Sharp model R-369 T) and irradiated for 20 min at a power of 550 W. Afterwards, the product was rinsed with deionised water six times and then completely dried at 80°C. The MWCNTs were denoted as functionalised MWCNTs (f-MWCNTs) after this process. The surface areas of the f-MWCNTs dramatically increased to 357.6 m^2^/g after the functionalisation process. Greater MWCNT surface area recorded after functionalisation has been associated with the increase of functional groups on the nanotube surface [39].

Preparation of TiO_2_/MWCNTs nanocatalysts involved the dispersion of f-MWCNTs in ethanol (pH = 2) and sonicated for 1 h. Then, approximately 561 μL of titanium isopropoxide (TTIP) was added dropwise to the suspension over a period of 20 min under vigorous stirring. Notably, under acidic conditions, the TiO_2_ surface contains positive charges due to the presence of ≡Ti-OH_2_^+^ groups [40], which enhance the adhesion characteristics on the MWCNTs' surface.

The amount of TTIP precursor represented a TiO_2_/f-MWCNT weight ratio of 50%. The mixture was then placed inside the modified microwave oven and irradiated for 5 min at a power of 550 W under continuous stirring. This suspension was subsequently dried at 100°C in a drying oven and then calcined at 500°C in air for 1 h to prepare the hybrid nanocatalysts.

The crystalline structure of the TiO_2_/MWCNTs nanocatalyst was characterised using X-ray powder diffraction (XRD) (Bruker D8 Advance, Karlsruhe, Germany) equipped with a Cu Kα radiation source operated at 40 kV and 40 mA. The powder morphology was determined by field-emission scanning electron microscopy (FE-SEM; SUPRA 55VP, Carl Zeiss, Jena, Germany) and transmission electron microscopy (TEM; Philips CM12, Amsterdam, The Netherlands; operated at 80 kV) studies. In addition, a Brunauer-Emmett-Teller (BET) (Micromeritics, ASAP 2020, Georgia, USA) was used to determine the surface area of the nanocatalyst.

The photocatalytic activity of the TiO_2_/MWCNTs nanocatalyst was evaluated by monitoring the degradation of methylene blue (MB) in an aqueous solution under irradiation with ultraviolet (UV) (VL-6.LC lamp) or visible light (VL) (commercial halogen tungsten lamp) using a custom-built setup. A small amount (1 mg) of the sample was suspended in 100 ml of aqueous MB solution with a concentration of 10 ppm. Prior to illumination, the solution was sonicated for 10 min and placed in a dark room for 1 h, thus permitting equilibration of the adsorption–desorption of the dye on the nanocatalyst surface. The first sample (approximately 5 mL) solution was collected immediately and was taken as the initial MB concentration (*c*_0_). The solution was then continuously shaken at 200 rpm. Approximately 5 mL of the liquid was withdrawn every 20 min and immediately centrifuged to remove any suspended solids. To monitor the degradation of the MB, the clean solution was then analysed using a UV–Visible spectrometer (Perkin Elmer, Lambda 900 UV/Vis) in the range of 500–750 nm.

## Results and discussion

**Figure 1:**
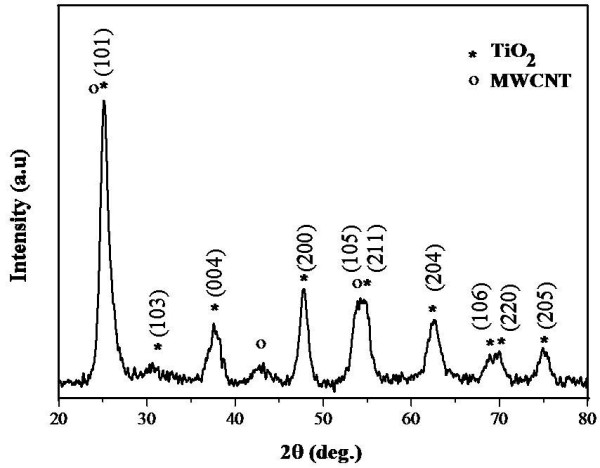
**X-ray diffractograms of the TiO**_**2**_**/MWCNT hybrids.**

The X-ray diffractogram of the synthesised TiO_2_/MWCNTs nanocatalysts showed the presence of several crystalline peaks, which are predominantly attributed to anatase TiO_2_ (Figure 1) [41]. The presence of this phase is due to the significantly high concentration of TiO_2_ in the material as well as weak X-ray scattering by MWCNTs. Most of the TiO_2_ peaks were broad with the calculated crystallite size of approximately 10 nm. The presence of MWCNTs was confirmed by the existence of a peak at a 2θ angle of 42.8°, whereas two other main peaks positioned at 26.1° and 53.6° overlapped substantially with TiO_2_ peaks.

**Figure 2:**
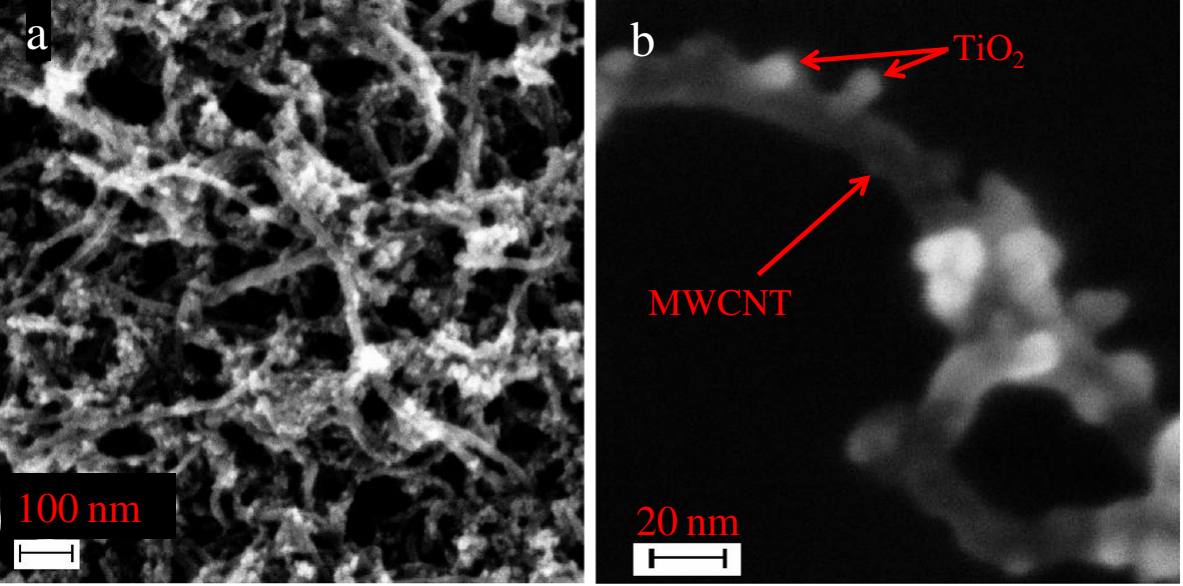
**FE-SEM micrograph of MWCNTs decorated with TiO**_**2 **_**nanoparticles. (a)** low magnification (×50,000) and **(b)** high magnification (×200,000).

Figure 2 depicts the FE-SEM images of the TiO_2_/MWCNTs nanocatalyst. The TiO_2_ nanoparticles that were produced *in situ* exhibit a mean particle size of approximately 10 nm. The images illustrate that the TiO_2_ nanoparticles were well attached to the MWCNTs. In addition, the TiO_2_/MWCNTs were well dispersed, although a few tangles were observed due to the length of the MWCNTs.

**Figure 3:**
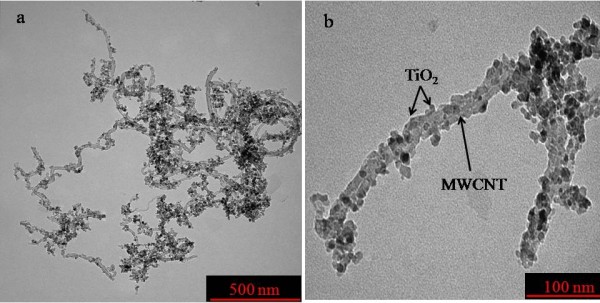
**TEM images of MWCNTs decorated with TiO**_**2 **_**nanoparticles: (a) low magnification and (b) high magnification.**

This result was further confirmed by TEM micrographs of the TiO_2_/MWCNT nanocatalyst (Figure 3). The TiO_2_ nanoparticles existed in the size of approximately 10 nm which was in good agreement with the calculated crystallite size. The interface between the MWCNTs and TiO_2_ is clearly observed, which confirms that the TiO_2_ nanoparticles were well attached to the surface of the MWCNTs. Compared to previous studies in which the synthetic methods required several hours for the attachment of TiO_2_[42-44], the procedures employed here required only a few minutes, which represents a clear and significant advantage of our method. Since the surface of MWCNT is well decorated with TiO_2_ nanoparticles, the inner core was barely visible. Apparently, the diameter of the decorated MWCNTs was increased compared to that of the bare MWCNTs. A similar finding was reported by other researchers using hydrothermal [45] and sol-gel [46] methods.

**Figure 4:**
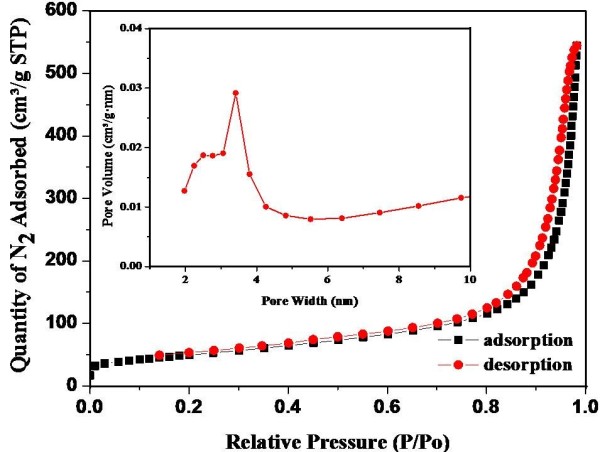
**N**_**2 **_**adsorption-desorption isotherms and the pore diameter distribution (inset) of the TiO**_**2**_**/MWCNTs nanocatalysts.**

Typical N_2_ adsorption and desorption isotherms for the hybrid nanocatalyst are shown in Figure 4. The surface area of the nanocatalyst was found to be 241.3 m^2^/g which is greater than previous reports [47,48]. This observation suggested that the f-MWCNTs' surface might be blocked by the attachment of TiO_2_ nanoparticles. It also suggested that the presence of the MWCNTs increased the specific surface area of the nanocatalyst, which led to its higher adsorptive ability.

At low pressures, the surface is only partially occupied by the gas, whereas the monolayer is filled and the isotherm reaches a plateau at higher pressures. Based on these results, the nanocatalyst can be ascribed to a type IV adsorption isotherm according to the IUPAC classification scheme; this result suggests that the structure of the nanocatalyst is mesoporous.

The pore size distribution of the TiO_2_/MWCNTs nanocatalysts was investigated based on the Barrett-Joyner-Halenda process (inset in Figure 4). The material shows bimodal mesopore size distributions, i.e. narrow mesopores with peak pore diameters of approximately 2.5 nm and larger mesopores with peak pore diameters of approximately 3.4 nm [49].

**Figure 5:**
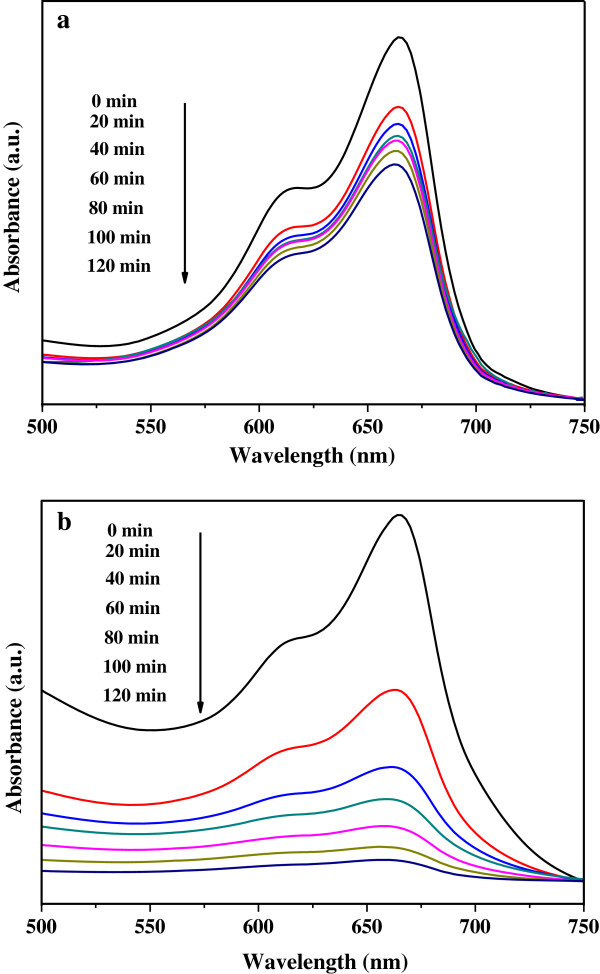
**UV-Vis absorption spectra of MB solutions after photocatalysis for different illumination times.** With TiO_2_/MWCNT nanocatalysts under UV **(a)** and VL **(b)** irradiation.

The change in the maximum absorption of MB illuminated under UV or VL over the TiO_2_/MWCNTs hybrid nanocatalyst material is shown in Figure 5. As the illumination time increased, the intensities of the maximum absorption peaks decreased, which suggests progressive decomposition of MB. Under both illuminations, the fastest rate of MB degradation was observed during the first 20 min, and the rate then gradually decreased as time increased. However, MB degraded slower under irradiation with UV light (Figure 5a) than under irradiation with VL (Figure 5b). This observation suggests that the photocatalytic activity of the hybrid nanocatalyst was enhanced under irradiation with visible light.

**Figure 6:**
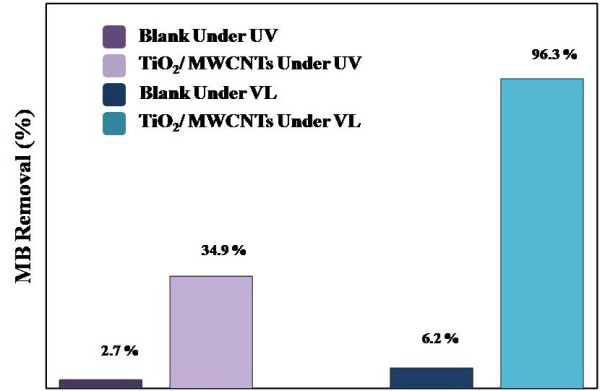
**Photocatalytic degradation behaviours of MB over TiO**_**2**_**/MWCNT nanocatalysts under UV and VL irradiation.**

The percentage of MB removed after 120 min under UV and VL illumination is presented in Figure 6. Under both illumination conditions, an insignificant reduction of the blank MB (without the catalyst) was observed in the solution, which confirms that MB cannot be degraded without a catalyst. Under UV illumination, the solution with the TiO_2_/MWCNTs nanocatalyst removed 34.9% of the MB. The surprising result was obtained while 96.3% of MB was removed when the solution was irradiated with VL. This result indicates that the TiO_2_/MWCNTs nanocatalyst prepared in this work is extremely photoactive under irradiation with VL, which results from that MWCNTs can act as a photosensitising agent when excited under visible-light irradiation [50,51]. Importantly, although only 1 mg of nanocatalyst was used in this work, the MB degradation was more extensive than that reported previously [52-54], indicating a promising future of this nanocatalyst.

## Conclusions

We successfully synthesised a hybrid nanocatalyst by attaching TiO_2_ nanoparticles onto MWCNTs at a weight ratio of 50% using a novel one-step method. The microstructure and morphology of the hybrid nanocatalyst were characterised by XRD, FESEM and TEM. The results showed that the anatase-phase TiO_2_ nanoparticles were attached to the surface of the MWCNTs. The BET surface area of the MWCNTs decreased after the TiO_2_ was attached to their surface. In addition, the efficiency of MB degradation under visible light was substantially greater compared to the efficiency under ultraviolet irradiation. These results indicate that MWCNTs can act as a photosensitiser agent and are excited under visible-light irradiation.

## Competing interests

The authors declare that they have no competing interests.

## Authors’ contributions

FKMA, MHHJ and SR participated in the design of the study. FKMA modified the microwave and prepared and characterized the hybrid nanocatalyst. NJR and AAU participated in the analysis of the experimental results. MAY gave his help on the BET measurement and analysis. FKMA and MHHJ jointly prepared the manuscript. All authors read and approved the final manuscript.

## References

[B1] Murugan K Rao TN Gandhi AS Murty B Effect of aggregation of methylene blue dye on TiO_2_ surface in self-cleaning studies Catal Commun 2010 8 518 521 10.1016/j.catcom.2009.12.007

[B2] Schäfer A Nghiem L Waite T Removal of the natural hormone estrone from aqueous solutions using nanofiltration and reverse osmosis Environ Sci Technol 2003 8 182 188 10.1021/es0102336 12542309

[B3] Zhang M Wang J Fu H Preparation and photocatalytic activity of nanocrystalline TiO_2_ with uniform shape and size J Mater Process Technol 2008 8 274 278 10.1016/j.jmatprotec.2007.08.037

[B4] Yasar A Tabinda AB Anaerobic treatment of industrial wastewater by UASB reactor integrated with chemical oxidation processes; an overview Pol J Environ Stud S 2010 8 1051 1061

[B5] Malato S Fernández-Ibáñez P Maldonado M Blanco J Gernjak W Decontamination and disinfection of water by solar photocatalysis: recent overview and trends Catal Today 2009 8 1 59 10.1016/j.cattod.2009.06.018

[B6] Chaudhari K Bhatt V Bhargava A Seshadri S Combinational system for the treatment of textile waste water: a future perspective Asian J Water Environ Pollut 2011 8 127 136

[B7] Hai FI Yamamoto K Fukushi K Hybrid treatment systems for dye wastewater Crit Rev Env Sci Technol 2007 8 315 377 10.1080/10643380601174723

[B8] Rauf M Meetani M Hisaindee S An overview on the photocatalytic degradation of azo dyes in the presence of TiO_2_ doped with selective transition metals Desalination 2011 8 13 27 10.1016/j.desal.2011.03.071

[B9] Akpan U Hameed B Parameters affecting the photocatalytic degradation of dyes using TiO_2_-based photocatalysts: a review J Hazard Mater 2009 8 520 529 10.1016/j.jhazmat.2009.05.039 19505759

[B10] Toor AP Verma A Jotshi C Bajpai P Singh V Photocatalytic degradation of direct yellow 12 dye using UV/TiO_2_ in a shallow pond slurry reactor Dyes Pigm 2006 8 53 60 10.1016/j.dyepig.2004.12.009

[B11] Liu K Zhu L Jiang T Sun Y Li H Wang D Mesoporous TiO_2_ micro-nanometer composite structure: synthesis, optoelectric properties, and photocatalytic selectivity Int J Photoenergy 2012 8 1 14

[B12] Robert D Malato S Solar photocatalysis: a clean process for water detoxification Sci Total Environ 2002 8 85 97 10.1016/S0048-9697(01)01094-4 12150445

[B13] Gaya UI Abdullah AH Heterogeneous photocatalytic degradation of organic contaminants over titanium dioxide: a review of fundamentals, progress and problems J Photochem Photobiol, C 2008 8 1 12 10.1016/j.jphotochemrev.2007.12.003

[B14] Hernández-Alonso MD Fresno F Suárez S Coronado JM Development of alternative photocatalysts to TiO_2_: challenges and opportunities Energy Environ Sci 2009 8 1231 1257 10.1039/b907933e

[B15] Xiang Q Yu J Jaroniec M Nitrogen and sulfur co-doped TiO2 nanosheets with exposed 001 facets: synthesis, characterization and visible-light photocatalytic activity Phys Chem Chem Phys 2011 8 4853 4861 10.1039/c0cp01459a 21103562

[B16] Yu H Irie H Shimodaira Y Hosogi Y Kuroda Y Miyauchi M Hashimoto K An efficient visible-light-sensitive Fe (III)-grafted TiO_2_ photocatalyst J Phys Chem C 2010 8 16481 16487 10.1021/jp1071956

[B17] Yao W Zhang B Huang C Ma C Song X Xu Q Synthesis and characterization of high efficiency and stable Ag_3_PO_4_/TiO_2_ visible light photocatalyst for the degradation of methylene blue and rhodamine B solutions J Mater Chem 2012 8 4050 4055 10.1039/c2jm14410g

[B18] Hashimoto K Irie H Fujishima A TiO_2_ photocatalysis: a historical overview and future prospects AAPPS Bull 2007 8 8269 8285

[B19] Zhou W Pan K Qu Y Sun F Tian C Ren Z Tian G Fu H Photodegradation of organic contamination in wastewaters by bonding TiO_2_/single-walled carbon nanotube composites with enhanced photocatalytic activity Chemosphere 2010 8 555 561 10.1016/j.chemosphere.2010.08.059 20851455

[B20] Clemens P Wei X Wilson BL Thomas RL Anatase titanium dioxide coated single wall carbon nanotubes manufactured by sonochemical-hydrothermal Jour Compos Mater 2013 8 21 32

[B21] Vatanpour V Madaeni SS Moradian R Zinadini S Astinchap B Novel antibifouling nanofiltration polyethersulfone membrane fabricated from embedding TiO_2_ coated multiwalled carbon nanotubes Sep Purif Technol 2012 8 69 82

[B22] Zhao D Yang X Chen C Wang X Enhanced photocatalytic degradation of methylene blue on multiwalled carbon nanotubes-TiO_2_ J Colloid Interface Sci 2013 8 1 6 10.1016/j.jcis.2013.02.017 23489609

[B23] Min Y Zhang K Zhao W Zheng F Chen Y Zhang Y Enhanced chemical interaction between TiO_2_ and graphene oxide for photocatalytic decolorization of methylene blue Chem Eng J 2012 8 203 210

[B24] Zhao D Sheng G Chen C Wang X Enhanced photocatalytic degradation of methylene blue under visible irradiation on graphene@TiO_2_ dyade structure Appl Catal, B 2012 8 303 308

[B25] Zhang Q Li C Li T Rapid photocatalytic degradation of methylene blue under high photon flux UV irradiation: characteristics and comparison with routine low photon flux Int J Photoenergy 2012 8 1 7

[B26] Liu J An T Li G Bao N Sheng G Fu J Preparation and characterization of highly active mesoporous TiO_2_ photocatalysts by hydrothermal synthesis under weak acid conditions Microporous Mesoporous Mater 2009 8 197 203 10.1016/j.micromeso.2009.05.009

[B27] Réti B Németh K Németh Z Mogyorósi K Markó K Erdőhelyi A Dombi A Hernadi K Photocatalytic measurements of TiO_2_/MWCNT catalysts having different surface coverage Phys Status Solidi B 2011 8 2475 2479 10.1002/pssb.201100080

[B28] Zhang K MENG Z OH W Degradation of rhodamine B by Fe-carbon nanotubes/TiO_2_ composites under UV light in aerated solution Chin J Catal 2010 8 751 758 10.1016/S1872-2067(09)60084-X

[B29] Hu C Zhang R Xiang J Liu T Li W Li M Duo S Wei F Synthesis of carbon nanotube/anatase titania composites by a combination of sol–gel and self-assembly at low temperature J Solid State Chem 2011 8 1286 1292 10.1016/j.jssc.2011.03.040

[B30] Xie Y Qian H Zhong Y Guo H Hu Y Facile low-temperature synthesis of carbon nanotube/TiO_2_ nanohybrids with enhanced visible-light-driven photocatalytic activity Int J Photoenergy 2012 8 1 6

[B31] Li Z Gao B Chen GZ Mokaya R Sotiropoulos S Li Puma G Carbon nanotube/titanium dioxide (CNT/TiO_2_) core–shell nanocomposites with tailored shell thickness, CNT content and photocatalytic/photoelectrocatalytic properties Appl Catal, B 2011 8 50 57

[B32] Yang H Wu S Duan Y Fu X Wu J Surface modification of CNTs and enhanced photocatalytic activity of TiO_2_ coated on hydrophilically modified CNTs Appl Surf Sci 2012 8 3012 3018 10.1016/j.apsusc.2011.11.029

[B33] Wang GJ Lee MW Chen YH A TiO_2_/CNT coaxial structure and standing CNT array laminated photocatalyst to enhance the photolysis efficiency of TiO_2_ Photochem Photobiol 2008 8 1493 1499 10.1111/j.1751-1097.2008.00374.x 18513231

[B34] Mahmood MA Dutta J Microwave assisted hydrothermal synthesis of zinc hydroxystannate films on glass substrates J Sol-gel Sci Technol 2012 8 495 504 10.1007/s10971-012-2754-2

[B35] Caddick S Fitzmaurice R Microwave enhanced synthesis Tetrahedron 2009 8 3325 3355 10.1016/j.tet.2009.01.105

[B36] He Z Yang S Ju Y Sun C Microwave photocatalytic degradation of rhodamine B using TiO_2_ supported on activated carbon: mechanism implication J Environ Sci 2009 8 268 272 10.1016/S1001-0742(08)62262-7 19402433

[B37] Cui L Hui K Hui K Lee S Zhou W Wan Z Thuc CNH Facile microwave-assisted hydrothermal synthesis of TiO_2_ nanotubes Mater Lett 2012 8 175 178

[B38] Jumali MHH Alosfur F Radiman S Umar AA Dressing of MWCNTs with TiO_2_ nanoparticles using modified microwave method Adv Mat Res 2012 8 228 231

[B39] Carabineiro SA Pereira MF Pereira JN Caparros C Sencadas V Lanceros-Mendez S Effect of the carbon nanotube surface characteristics on the conductivity and dielectric constant of carbon nanotube/poly (vinylidene fluoride) composites Nanoscale Res Lett 2011 8 1 5 10.1186/1556-276X-6-302 PMC3211369 21711832

[B40] Choi W Pure and modified TiO_2_ photocatalysts and their environmental applications Catal Surv Asia 2006 8 16 28 10.1007/s10563-006-9000-2

[B41] Zaine SNA Mastan AAK Shaik Ahmedullah S Mohamed NM Ramli A Ahmad IA Synthesis and characterization of pure anatase TiO_2_ aggregates J Appl Sci 2011 8 1326 1330

[B42] Sánchez M Guirado R Rincón M Multiwalled carbon nanotubes embedded in sol–gel derived TiO_2_ matrices and their use as room temperature gas sensors J Mater Sci Mater Electron 2007 8 1131 1136 10.1007/s10854-007-9144-5

[B43] Rakhi R Sethupathi K Ramaprabhu S Field emission properties of metal encapsulated and metal-oxide dispersed multi-walled carbon nanotube nanocomposites J Nano Energy Power Res 2011 8 57 64 10.1166/jnepr.2011.1008

[B44] Yu J Ma T Liu S Enhanced photocatalytic activity of mesoporous TiO_2_ aggregates by embedding carbon nanotubes as electron-transfer channel Phys Chem Chem Phys 2010 8 3491 3501 21173966 10.1039/c0cp01139h

[B45] Németh Z Dieker C Kukovecz Á Alexander D Forró L Seo JW Hernadi K Preparation of homogeneous titania coating on the surface of MWNT Compos Sci Technol 2011 8 87 94 10.1016/j.compscitech.2010.10.017

[B46] Chen L Pang X Yu G Zhang J In-situ coating of MWNTs with sol–gel TiO_2_ nanoparticles Adv Mater Lett 2010 8 75 78 10.5185/amlett.2010.4117

[B47] Zhang K Zhang FJ Chen ML Oh WC Comparison of catalytic activities for photocatalytic and sonocatalytic degradation of methylene blue in present of anatase TiO_2_–CNT catalysts Ultrason Sonochem 2011 8 765 772 10.1016/j.ultsonch.2010.11.008 21146437

[B48] Song C Chen P Wang C Zhu L Photodegradation of perfluorooctanoic acid by synthesized TiO_2_–MWCNT composites under 365 nm UV irradiation Chemosphere 2012 8 853 859 10.1016/j.chemosphere.2011.11.034 22172634

[B49] Yu J Fan J Cheng B Dye-sensitized solar cells based on anatase TiO_2_ hollow spheres/carbon nanotube composite films J Power Sources 2011 8 7891 7898 10.1016/j.jpowsour.2011.05.014

[B50] Woan K Pyrgiotakis G Sigmund W Photocatalytic carbon-nanotube–TiO_2_ composites Adv Mater 2009 8 2233 2239 10.1002/adma.200802738

[B51] Huang HC Huang GL Chen HL Lee YD Immobilization of TiO_2_ nanoparticles on carbon nanocapsules for photovoltaic applications Thin Solid Films 2006 8 203 207

[B52] Lu SY Tang CW Lin YH Kuo HF Lai YC Tsai MY Ouyang H Hsu WK TiO_2_-coated carbon nanotubes: a redshift enhanced photocatalysis at visible light Appl Phys Lett 2010 8 231915 231913 10.1063/1.3454908

[B53] Jiang G Zheng X Wang Y Li T Sun X Photo-degradation of methylene blue by multi-walled carbon nanotubes/TiO_2_ composites Powder Technol 2011 8 465 469 10.1016/j.powtec.2010.11.029

[B54] Tian L Ye L Deng K Zan L TiO_2_/carbon nanotube hybrid nanostructures: solvothermal synthesis and their visible light photocatalytic activity J Solid State Chem 2011 8 1465 1471 10.1016/j.jssc.2011.04.014

